# Retraction: Sung et al. Expression of SARS-CoV-2 Spike Protein Receptor Binding Domain on Recombinant *B. subtilis* on Spore Surface: A Potential COVID-19 Oral Vaccine Candidate. *Vaccines* 2022, *10*, 2

**DOI:** 10.3390/vaccines10111852

**Published:** 2022-11-01

**Authors:** Johnny Chun-Chau Sung, Ying Liu, Kam-Chau Wu, Man-Chung Choi, Chloe Ho-Yi Ma, Jayman Lin, Emily Isabel Cheng He, David Yiu-Ming Leung, Eric Tung-Po Sze, Yusuf Khwaja Hamied, Dominic Man-Kit Lam, Keith Wai-Yeung Kwong

**Affiliations:** 1 Research Department, DreamTec Cytokines Limited, Hong Kong, China; 2 Oristry BioTech (HK) Limited, Hong Kong, China; 3 Meserna Therapeutic (HK) Limited, Hong Kong, China; 4 L&L Immunotherapy Company Limited, Hong Kong, China; 5 Zentrogene Bioscience Laboratory Limited, Hong Kong, China; 6 Health Plus Laboratory Limited, Hong Kong, China; 7 School of Science and Technology, Hong Kong Metropolitan University, Hong Kong, China; 8 Cipla Limited, Mumbai 400013, India; 9 Torsten Wiesel International Research Institute, Sichuan University, Chengdu 610017, China

The journal retracts the article “Expression of SARS-CoV-2 Spike Protein Receptor Binding Domain on Recombinant *B. subtilis* on Spore Surface: A Potential COVID-19 Oral Vaccine Candidate” [1].

Following publication, concerns were brought to the attention of the publisher regarding the ethical approval not granted from Hong Kong Metropolitan University (HKMU). In adherence to our complaints procedure, an investigation was conducted, and the article is therefore retracted. This retraction was approved by the Publication Ethics Committee and the Editor-in-Chief.

The authors agreed to this retraction.
